# Murine myoblast migration: influence of replicative ageing and nutrition

**DOI:** 10.1007/s10522-017-9735-3

**Published:** 2017-11-07

**Authors:** Alexander D. Brown, Graeme L. Close, Adam P. Sharples, Claire E. Stewart

**Affiliations:** Stem Cells, Ageing & Molecular Physiology Unit, Research Institute for Sport and Exercise Sciences (RISES), School of Sport and Exercise Sciences, Liverpool John Moores University, Liverpool, UK

**Keywords:** Myoblast, HMB, Leucine, PI3K, ERK, mTOR, Damage, Ageing

## Abstract

Cell migration is central to skeletal muscle repair following damage. Leucine and β-Hydroxy β-methylbutyric acid (HMB) are supplements consumed for recovery from muscle damaging exercise in humans, however, their impact on muscle cell migration with age is not yet understood. We hypothesised that replicatively aged (“aged”; P46–P48) myoblasts would be less efficient at basal and supplemented repair versus parental controls (“control”; P12–P16). Aged and control myoblasts were scratch-damaged and migration velocity, directionality and distance assessed over 48 h in the absence and presence of leucine (10 mM) or HMB (10 mM) ± PI3K/Akt (LY294002 10 μM), ERK (PD98059 5 μM) or mTOR (rapamycin 0.5 μM) inhibition. Opposing our hypothesis, aged cells displayed increased velocities, directionality and distance migrated (P < 0.001) versus control. Leucine and HMB significantly increased (P < 0.001) the same parameters in control cells. The supplements were with smaller, albeit significant impact on aged cell velocity (P < 0.001) and in the presence of HMB only, distance (P = 0.041). Inhibitor studies revealed that, PI3K and ERK activation were essential for velocity, directionality and migration distance of aged cells in basal conditions, whereas mTOR was important for directionality only. While PI3K activation was critical for all parameters in control cells (P < 0.001), inhibition of ERK or mTOR improved, rather than reduced, control cell migration distance. Enhanced basal velocity, directionality and distance in aged cells required ERK and PI3K activation. By contrast, in control cells, basal migration was underpinned by PI3K activation, and facilitated by leucine or HMB supplementation, to migration levels seen in aged cells. These data suggest that replicatively aged myoblasts are not anabolically resistant per se, but are capable of efficient repair, underpinned by altered signaling pathways, compared with unaged control myoblasts.

## Introduction

During the human lifespan, a gradual loss of skeletal muscle mass and strength occurs, referred to as sarcopenia. While muscle mass and strength in young individuals can be preserved through nutritional supplementation, it is reported that muscle in older adults displays a level of anabolic resistance (Breen and Phillips 2011). The capacity of the muscle to regenerate following exercise induced muscle damage is reportedly impaired in ageing rodents and humans (Brooks and Faulkner 1988; Faulkner et al. 1991). It is reported that altered satellite cell behaviour may negatively impact not only on muscle mass and strength, but also on the muscle regeneration processes (Welle 2002; Shefer et al. 2006; Day et al. 2010; Bigot et al. 2015).

Recently, interest has arisen relating to the use of nutraceuticals to facilitate muscle growth. Data suggest old muscle may be anabolically resistant and require higher concentrations of protein to elicit a hypertrophic response versus young muscle (Breen and Phillips 2011). Leucine, an essential amino acid, is reportedly a potent anabolic agent (Koopman et al. 2006) and is also consumed following damaging exercise, with the aim to improve muscle regeneration (Farup et al. 2014). Recent studies have investigated the effects of leucine administration on myoblast fusion (Areta et al. 2014; Dai et al. 2015) and demonstrated that increasing leucine in a dose responsive manner (5 and 16.5 mM) stimulated the mTOR signaling pathway and the phosphorylation of P70S6K, resulting in significantly increased myoblast fusion. Furthermore, in young recreationally active males, whey protein, which contains high doses of leucine (8 g per 100 g), increased muscle satellite cell number at 48 h post eccentric damage, compared with control (Farup et al. 2014).

Hydroxy β-methylbutyric acid (HMB), a metabolite of leucine, is increasing in popularity as an ergogenic aid for muscle recovery and regeneration. HMB studies in human myoblasts and rodents demonstrate positive effects on satellite cell proliferation, differentiation and survival, following MAPK/ERK and PI3K/Akt activation (Kornasio et al. 2009; Vallejo et al. 2016). Supplementation of human myoblasts with HMB (0–85 mM) stimulated cell proliferation via the MAPK/ERK pathway and induced differentiation via the PI3K/Akt pathway (Kornasio et al. 2009). Further studies by Vallejo et al. (2016) investigated the impact of HMB on C_2_C_12_ myoblasts (25–125 µM) and on the contractile force of ageing murine soleus muscle (514 mg/kg). HMB treatment increased C_2_C_12_ myoblast proliferation and myoblast viability. In mice, HMB prolonged force generation and reduced the amount of time for peak muscle contraction following damage (Vallejo et al. 2016). Together, these studies indicated that leucine and HMB could impact positively on muscle differentiation, survival and function.

Adequate skeletal muscle mass and function are essential in supporting human health and well-being [reviewed in (Sharples et al. 2015)]. However, the molecular regulators of skeletal muscle cell migration are relatively understudied, despite the fact that skeletal muscle has a remarkable ability to regenerate. Understanding the signaling pathways that regulate myoblast migration, direction and velocity is therefore important in advancing capacity to promote skeletal muscle regeneration. Evidence exists supporting the role of the Rho family, in regulating satellite cell migration (Raftopoulou and Hall 2004). Upstream of the Rho family is the PI3K/Akt pathway, which we demonstrated, when inhibited, resulted in impaired myoblast migration (Dimchev et al. 2013). Furthermore, the MAPK/ERK pathway is also reportedly involved in efficient myoblast migration, albeit findings are somewhat equivocal (Leloup et al. 2007; Ranzato et al. 2009; Al-Shanti et al. 2011).

Given a global drive to reduce/refine animal research, relevant cell models are required to inform future in vivo studies. To this end, we have developed a myoblast model, with application to ageing muscle cell behavior (Sharples et al. 2011). Using a process of replicative ageing, C_2_C_12_ murine skeletal muscle cells were subjected to 58 population doublings versus parental control and were reported to display impaired differentiation both in 2-D and 3-D models (Sharples et al.^2011^, ^2012^; Deane et al. 2013). While these cells have been extensively characterised with regards to hypertrophy and atrophy and compare well with replicatively aged human cells (Bigot et al. 2008), human and rodent cells isolated from ageing muscle (Lees et al. 2006; Bigot et al. 2008; Léger et al. 2008; Lees et al. 2009; Pietrangelo et al. 2009) and muscle biopsy tissue derived from older individuals (Welle et al. 2003; Léger et al. 2008), little research has focused on their ability to repair damage. Furthermore, although the potential of nutraceuticals in muscle preservation is being avidly investigated (Phillips et al. 2009), the question remaining to be challenged is whether nutraceuticals elicit a beneficial impact on muscle cell migration and repair and whether this is compromised with ageing.

Therefore, the goal of this study is to investigate the impact of leucine and HMB on control and aged skeletal muscle cell repair. The objectives are: 1. To determine the migration capacity of replicatively aged (but not senescent) C_2_C_12_ skeletal muscle cells versus controls that have not undergone any population doublings relative to aged cells. 2. To investigate the impact of the nutritional supplements leucine and HMB on migration capacity and; 3. To begin to determine relevant signaling pathways (PI3K, ERK and mTOR) that may be important for successful migration and wound closure. We hypothesised that: (1) replicatively aged (P46–P48; ‘aged’) myoblasts would be less efficient at damage repair versus unaged controls (P12–P16, ‘control’); (2) leucine and HMB would increase the migration potential in control but not aged cells and; (3) that the PI3K and ERK, but not mTOR (given its critical role in myoblast fusion) pathways would be required for effective migration in both models.

## Methods

### Cell culture

All cell culture procedures were conducted using a Kojair Biowizard Silverline class II hood (Kojair, Vippula, Finland). Commercially available C_2_C_12_ mouse skeletal myoblasts were purchased from ATCC and passages 12–16 (referred to as ‘control’) and passages 44–48 (replicative aged and referred to as ‘aged’; (130–140 population doublings)) were used in this study. The cells were incubated in a HERAcell 150i incubator (Thermo Scientific, Cheshire, UK) at: 5% CO_2_ and 37 °C. Cells were resuscitated from liquid nitrogen storage and seeded onto gelatinised T75 flasks (Nunc, Roskilde, Denmark) at 1 × 10^6^ cells/ml in growth medium (GM) that consisted of: Dulbecco’s Modified Eagle Medium (DMEM), 10% heat-inactivated fetal bovine serum, 10% heat-inactivated newborn calf serum, 2 mM l-glutamine, and 1% penicillin–streptomycin.

### Damage protocol and cell treatments

Once 80% confluency was attained, cells were trypsinized, counted and seeded at 100,000 cells/ml on gelatinised six or twelve well plates (Nunc, Roskilde, Denmark) and grown to 80% confluence. Cells were washed once with PBS prior to an established in vitro wound/repair model to assess migration in myoblasts via applying a damaging scratch to monolayer cells as previously reported by our group (Dimchev et al. 2013; Owens et al. 2015). Cells were washed twice with PBS to remove any debris, prior to dosing in differentiation medium (DM containing: DMEM, 2% heat-inactivated horse serum, 2 mM l-glutamine, 1% penicillin–streptomycin), in the absence or presence of leucine (10 mM) or HMB (10 mM). Doses were selected following basal dose response studies (0–10 mM, data not shown). In addition, signaling pathways, in the absence or presence of leucine or HMB, were manipulated with: LY294002 (10 μM), inhibitor of PI3K signalling, PD98059 (5 μm), inhibitor of ERK signalling or rapamycin (0.5 μM) inhibitor of mTOR (Dimchev et al. 2013; Hatfield et al. 2015). For inhibitor studies, cells were allowed to quiesce for 30 min, in DM, prior to addition of respective inhibitors for 30 min, followed by supplements for up to 48 h. All cell experiments were repeated 3 times in duplicate.

### Wound healing assay and migration analysis

For the wound/repair healing assays, immediately following treatment, cells were incubated in a controlled live imaging environment (Leica DMB 6000; equipped with PeCon incubation and gas control system) at 37 and 5% CO_2_. Microscopic images were obtained from two points within each wound (using track and find), every 30 min for 48 h at ×10 magnification. For the analysis of cell migration dynamics, the directionality, accumulated distance and velocity were determined using TIF image stacks and Image J software (IBIDI, Munich, Germany). The manual tracking and chemotaxis plug-ins were installed which allowed for individual cell trajectory and migration to be analysed. The chemotaxis tool analyses the raw data from the manual tracking plug-in and provides quantitative data on cell directionality (arbitrary units), velocity (μm/min) and accumulated distance (μm).

### Cell fixation and preparation for flow cytometry

FLOW cytometry was performed to simultaneously assess multiple phosphoproteins in relevant cell samples (Schubert et al. 2009, Sharples et al. 2011). At ~ 80% confluence, the cells were washed, damaged and quiesced prior to dosing and harvest as detailed above. This 30 min quiescence timepoint was designated as time 0 h. The cells were either fixed at time 0 h or dosed with 10 mM leucine or HMB for 15, 60 and 120 min post damage. The cells were washed twice in PBS prior to trypsinisation, neutralisation and centrifugation at 775 g for 5 min at 4 °C. The supernatant was removed and the cells were fixed in 2% paraformaldehyde at room temperature for 60 min. The cells were centrifuged as above and re-suspended in 100% methanol. Cells were stored at − 20 °C until further analyses by FLOW. The cells were washed in FLOW buffer (PBS + 0.5% FBS) and centrifuged at 500 g for 5 min at 4 °C and re-suspended in FLOW buffer. The anti-human/mouse phospho-AKT (S473; APC; 675/25; 0.5 ug), anti-human/mouse phospho-ERK1/2 (T202/Y204; Alexafluor 488; 533/30; 0.03 ug) and anti-human/mouse phospho-mTOR (S2448; PerCP; 670/LP; 0.125 ug) antibodies (Thermo Fisher Scientific inc, Waltham, USA) were added to each sample and incubated at room temperature in the dark for 60 min. The cells were washed a further three times, and re-suspended in 200 μl FLOW buffer. The samples were analysed using flow cytometry on a BD Accuri C6 flow cytometer with BD CFlow^®^ Software, collecting 2000 events per sample. Fluorophores used in flow cytometry can emit photons of multiple energies and wavelengths, compensation of individual fluorescent antibodies in multiple detectors was performed to reduce spectral overlap. Forward scatter and side scatter gating was performed to ensure single populations of cells. Together these processes should reduce data skew and improve accuracy.

### Statistical analysis

SPSS Predictive Analytics Software (version 23; IBM) was used for all statistical analyses. Data were assessed and normal distribution confirmed. For the comparison of replicative aging (control vs. aged C_2_C_12_ cells) versus supplements (DM alone, leucine, HMB) a two by three-way ANOVA was used, where significant main effects and interactions were present, the Bonferroni post hoc pairwise comparisons test was used. For within test comparisons, either, independent t-tests, or one-way analysis of variance (ANOVA) was used. All data are presented as mean ± SD and significance as ≤ 0.05.

## Results

### Improved migration in replicatively aged versus unaged control C_2_C_12_ skeletal muscle cells

**Fig. 1:**
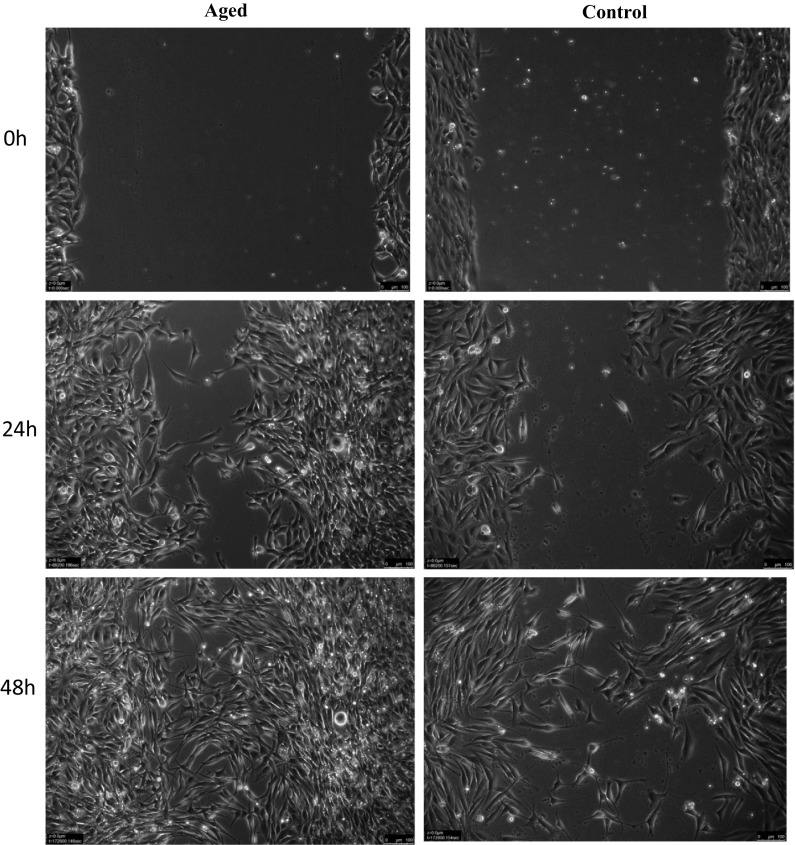
Images to show the difference between aged and control cells at 0, 24 and 48 h

**Fig. 2:**
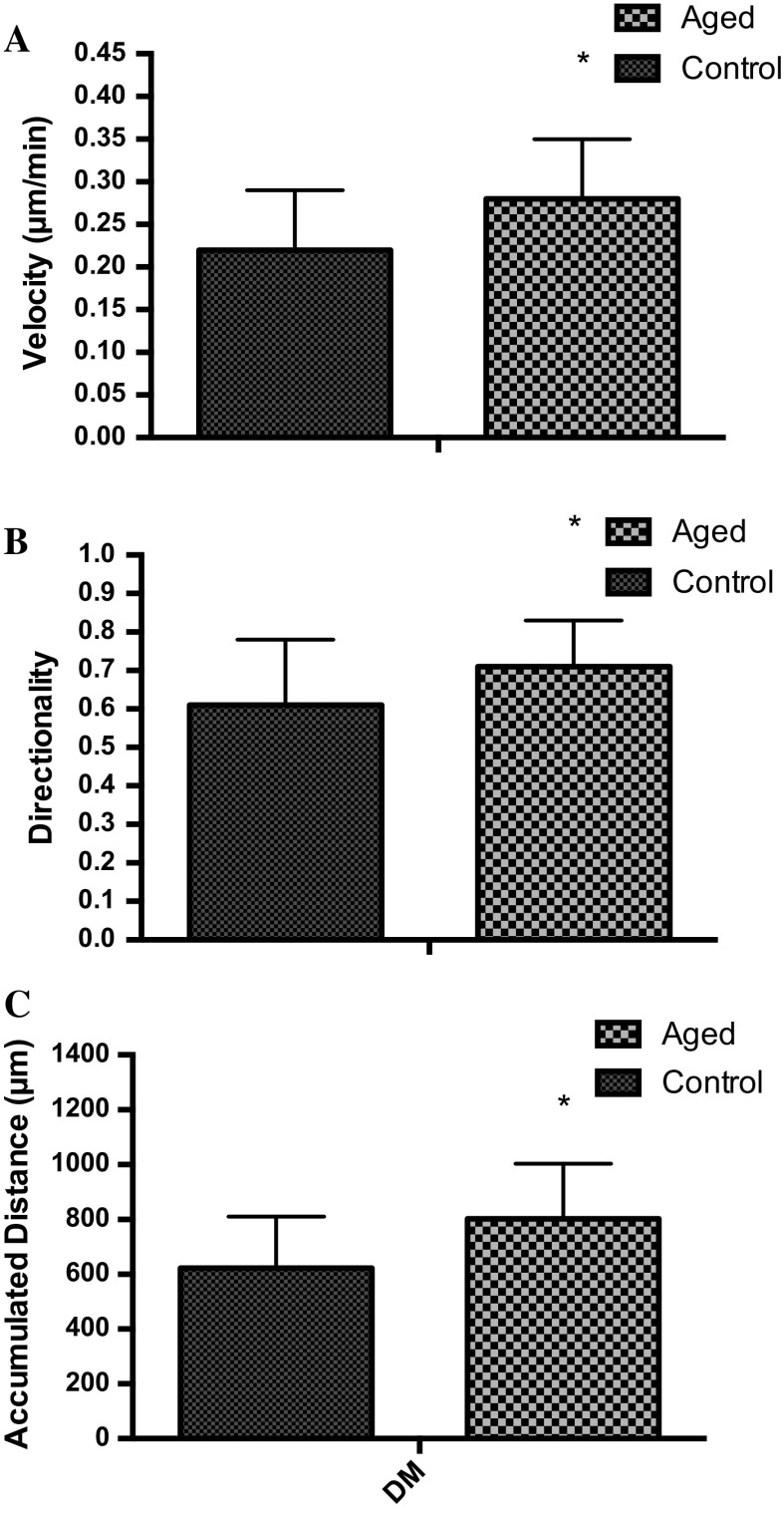
Bar charts illustrating the differences in cell velocity (**a**), directionality (**b**) and accumulated distance (**c**) between the aged and control over 48 h. The data is shown as mean with SD. Significance is set at P < 0.05, and the significance between aged versus control was indicated using *. The experiment consisted of 3 repeats all in duplicate

**Fig. 3:**
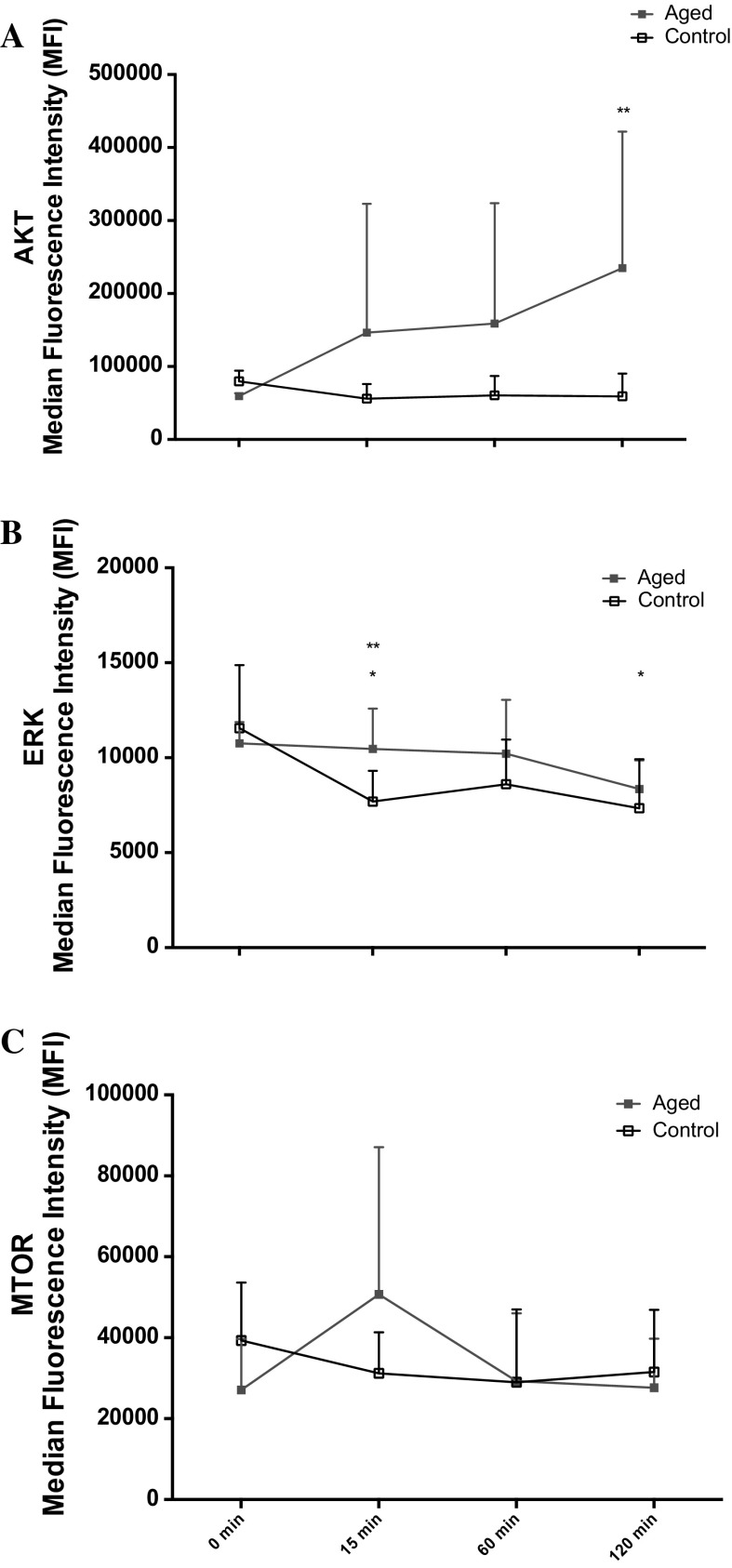
Line charts illustrating the differences in the phosphorylation of Akt (**a**), ERK (**b**) and mTOR (**c**) molecules between the aged and control over 120 min. The data is shown as mean with SD. Significance is set at P < 0.05. Significance was indicted versus 0 min (*) and versus corresponding time-point (**). The experiment consisted of 3 repeats all in duplicate

Following scratch damage, aged and control cell migration into the wound site was measured over 48 h (Fig. 1). In contrast to our hypothesis, under basal conditions in replicatively aged versus control cells respectively, there was a 1.27-fold increase in cell velocity (0.28 ± 0.07 μm/min^−1^ vs. 0.22 ± 0.07 μm/min^−1^; P < 0.001; Fig. 2a), a 1.27-fold increase in directionality (0.71 ± 0.12 vs. 0.61 ± 0.17; P < 0.001; Fig. 2b) and a 1.29-fold increase in overall migration distance (802 ± 202 μm vs. 622 ± 188 μm; P < 0.001; Fig. 2c). This increased migration in the aged cells was associated with altered phosphorylation of Akt, ERK, and mTOR (Fig. 3a, b, c, respectively). In aged cells ERK activation was significantly increased (P = 0.028) versus control cells at 15 min and while still elevated at 60 min, significance was not attained. Akt phosphorylation was not different between the two cell groups at 15 min. However, while Akt phosphorylation decreased in the unaged controls to 60 min where it plateaued to 120 min, it increased over the time course in the aged cells reaching significance (P = 0.047) versus control at 120 min. Finally, mTOR phosphorylation did not significantly change over the time course assessed in aged or control cells and no significant differences were observed between the two models.

**Fig. 4:**
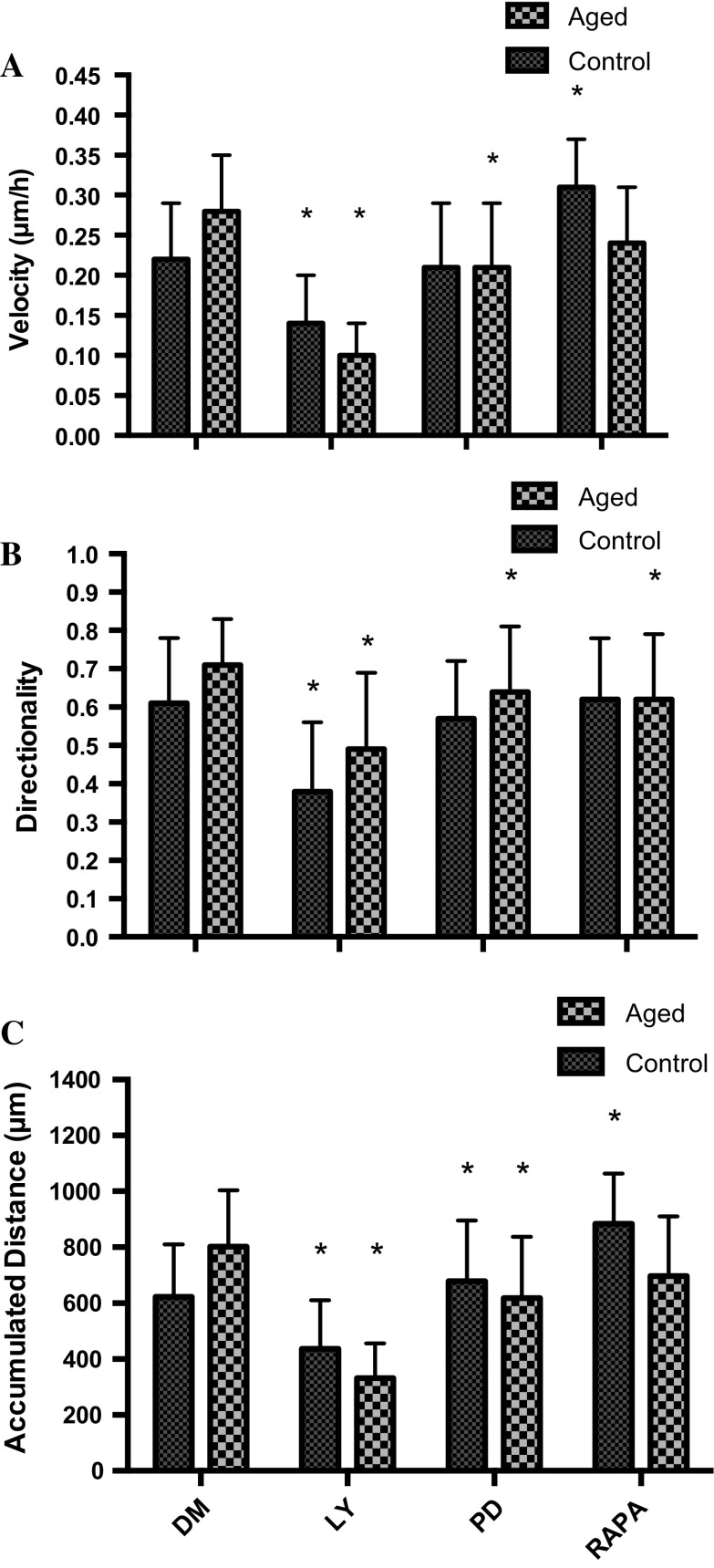
Bar charts illustrating the differences in cell velocity (**a**), directionality (**b**) and accumulated distance (**c**) in the aged and control versus LY294002, PD98059 and rapamycin. The data is shown as mean with SD. Significance is set at P < 0.05, and the significance between the inhibitors versus basal aged and control cells, was indicated using *. The experiment consisted of 3 repeats all in duplicate

Wishing to determine whether the altered signaling profiles evident between the cell models may impact on improved migration in the replicatively aged model, inhibitor studies were performed. Cell velocity was 2.8-fold (0.28 ± 0.07 μm/min^−1^ vs. 0.10 ± 0.04 μm/min^−1^; P < 0.001), directionality, 1.5-fold (0.71 ± 0.12 vs. 0.49 ± 0.20; P < 0.001) and overall migration distance, 2.4-fold (802 ± 202 μm vs. 332 ± 123 μm; P < 0.001) greater in the absence versus presence of the PI3K inhibitor, LY294002, respectively (Fig. 4). Similarly, when aged cells were incubated with the ERK inhibitor (PD98059), cell velocity was 1.3-fold (0.28 ± 0.07 μm/min^−1^ vs. 0.21 ± 0.08 μm/min^1^; P = 0.001), directionality, 1.1-fold (0.71 ± 0.12 vs. 0.64 ± 0.17; P < 0.001) and accumulated distance 1.3-fold (802 ± 202 μm vs. 618 ± 219 μm; P < 0.001) higher under control versus inhibitor conditions, respectively (Fig. 4). Indeed, in the presence of PD98059, migration potential of the aged cells was reduced to that of control cells, the latter under control conditions. Finally, under aged control versus mTOR inhibition (rapamycin administration), despite a small reduction in velocity versus untreated aged control (Fig. 4), significance was not attained (0.24 ± 0.07 μm/min^−1^ vs. 0.28 ± 0.07 μm/min^−1^). Compared with untreated aged control cells in the presence of rapamycin, directionality was significantly reduced by 1.15-fold (0.71 ± 0.12 vs. 0.62 ± 0.17; P < 0.001), however, this was not sufficient to significantly reduce overall migration distance (802 ± 202 μm vs. 697 ± 213 μm).

Having determined central roles for PI3K/Akt and ERK in all parameters of aged cell migration and mTOR in directionality, equivalent studies were performed in the control cell model (Fig. 4). Similar to the aged cells, compared with control, when incubated with PI3K/Akt inhibitor (LY294002), cell velocity was 1.6-fold (0.22 ± 0.07 μm/min^−1^ vs. 0.14 ± 0.06 μm/min^−1^, P < 0.001), directionality, 1.6-fold (0.61 ± 0.17 vs. 0.38 ± 0.18, P < 0.001) and accumulated migration distance, 1.45-fold (622 ± 188 μm vs. 437 ± 174 μm; P < 0.001) higher under control versus inhibitor conditions, respectively. By contrast, compared with control, in the presence of ERK inhibition via PD98059 administration, cell velocity (0.22 ± 0.07 μm/min^−1^ vs. 0.21 ± 0.08 μm/min^−1^) directionality (0.61 ± 0.17 vs. 0.57 ± 0.15) and overall migration distance (622 ± 188 μm vs. 678 ± 217 μm; P = 0.013), were not altered (Fig. 4). Therefore, while ERK inhibition reduced the migration potential of aged cells to control capabilities; it was without impact on control cell migration. Finally, in complete contrast to the aged cell model, compared with control, when control cells were incubated with mTOR inhibitor, rapamycin, cell velocity was 1.4-fold (0.22 ± 0.07 μm/min^−1^ vs. 0.31 ± 0.06 μm/min^−1^; P < 0.001) higher. Directionality, which was reduced in aged cells, was unaltered (0.61 ± 0.17 vs. 0.62 ± 0.16), however, in line with increased velocity in control cells with rapamycin, overall migration distance was significantly increased by 1.4-fold (622 ± 188 μm vs. 884 ± 180 μm; P < 0.001) versus control. Indeed, the enhanced migration potential of control cells in the presence of rapamycin now was equivalent to that of aged cells (velocity and distance) under control conditions (Fig. 4).

Under basal conditions, inhibition of PI3K using LY294002 resulted in a significant reduction in aged cell migration velocity vs. control cells (0.10 ± 0.04 μm/min^−1^ vs. 0.14 ± 0.06 μm/min^−1^; P < 0.001; Fig. 4a), significantly reduced control cell directionality versus aged (0.38 ± 0.18 vs. 0.49 ± 0.20; P < 0.001; Fig. 4b) and resulted in an overall reduction in migration distance of aged vs. unaged cells (332 ± 123 μm vs. 437 ± 174 μm; P < 0.001; Fig. 4c). By contrast, when administration of ERK inhibitor, PD98059, there were no significant differences between control and replicatively aged cell velocity (0.21 ± 0.08 μm/min^−1^ vs. 0.21 ± 0.08 μm/min^−1^; Fig. 4a). However, despite cell directionality being reduced in control versus aged cells basally (0.57 ± 0.15 vs. 0.64 ± 0.17; P = 0.001; Fig. 4b), overall migration distance was significantly lower in aged versus control cells with ERK inhibition (618 ± 219 μm vs. 678 ± 217 μm; P < 0.05; Fig. 4c). Finally, the impact of rapamycin on cell velocity was greater in control versus aged cells (0.31 ± 0.06 μm/min^−1^ vs. 0.24 ± 0.07 μm/min^−1^; P < 0.001; Fig. 4a) and despite no differences in cell directionality (Fig. 4b), rapamycin resulted in a significantly increased migration distance in control versus aged cells (884 ± 180 μm vs. 697 ± 213 μm; P < 0.001; Fig. 4c).

### Effect of leucine and HMB supplementation on rodent C_2_C_12_ skeletal muscle cells migration

**Fig. 5:**
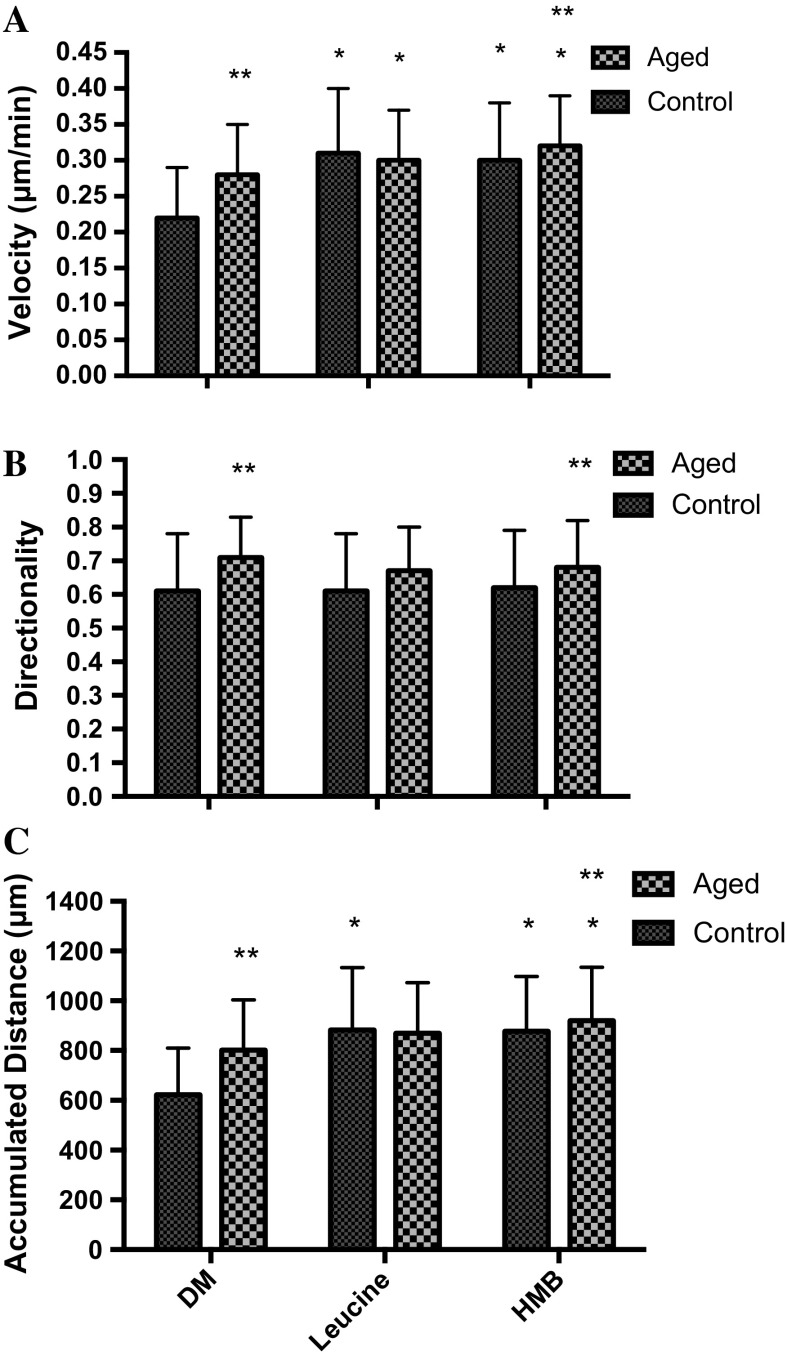
Bar charts illustrating the differences in cell velocity (**a**), directionality (**b**) and accumulated distance (**c**) in the aged and control versus leucine and HMB. The data is shown as mean with SD. Significance is set at P < 0.05, and the significance between the supplements versus basal aged and control cells, was indicated using *. The significance between the aged versus control was indicated using **. The experiment consisted of 3 repeats all in duplicate

To determine whether aged cell migration could be enhanced, treatment with leucine (10 mM) and HMB (10 mM) were performed. When supplemented with leucine, there was a small but significant 1.07-fold increase in aged cell velocity (0.30 ± 0.07 μm/min^−1^ vs. 0.28 ± 0.07 μm/min^−1;^ P < 0.001) and a small but significant 0.97-fold decrease in directionality (0.67 ± 0.13 vs. control 0.71 ± 0.12; P = 0.001) versus untreated control. Together, these changes were not sufficient to impact on overall migration distance, which was not different from untreated aged control (Fig. 5). In the presence of HMB, there was a small but significant 1.15-fold increase in velocity (0.32 ± 0.07 vs. 0.28 ± 0.07 μm/min^−1^; P < 0.001), no impact on directionality and an overall 1.15-fold increase in migration distance versus untreated aged control (921 ± 215 μm vs. 802 ± 202 μm; P = 0.041).

In contrast, to aged cells, it was hypothesised, that migration of control cells would be increased when supplemented with leucine or HMB. In the presence of leucine, cell velocity was significantly increased by 1.4-fold, (0.31 ± 0.09 μm/min^−1^; vs. 0.22 ± 0.07 μm/min^−1^; P < 0.001) versus untreated control, directionality was unaltered and overall migration distance increased by 1.4-fold (883 ± 250 μm vs. 622 ± 188 μm; P < 0.001). Indeed, in the presence of leucine, the enhanced velocity (0.31 ± 0.09 μm/min^−1^) and overall migration distance (883 ± 250 μm) of control cells were now not significantly different from velocity (0.30 ± 0.07 μm/min^−1^) and migration distance (871 ± 202 μm) of aged cells with leucine (Fig. 5). Similar to leucine, in control cells, HMB supplementation resulted in a 1.36-fold increase in velocity (0.30 ± 0.08 μm/min^−1^ vs. 0.22 ± 0.07 μm/min^−1^; P < 0.001) versus untreated control, no impact on directionality and a 1.41-fold increase in overall migration distance (878 ± 219 μm vs. 622 ± 188 μm; P < 0.001). Despite improved migration, in the presence of HMB, aged cells still displayed increased cell velocity (0.32 ± 0.07 μm/min^−1^ vs. 0.30 ± 0.08 μm/min^−1^; P < 0.05), directionality (0.68 ± 0.14 vs. 0.62 ± 0.17; P < 0.001) and overall migration distance (920 ± 215 μm vs. 878 ± 219 μm; P < 0.05) versus control cells, respectively (Fig. 5).

### Proposed signaling pathways that regulate cell migration

**Fig. 6:**
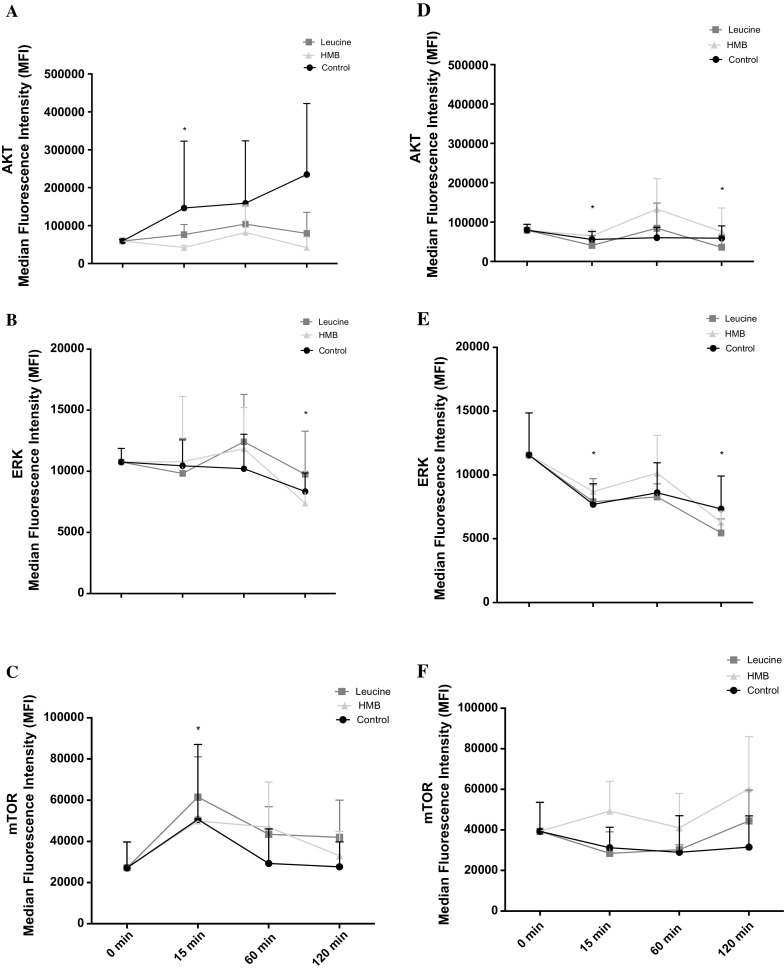
Line charts illustrating the differences in the phosphorylation of aged Akt (**a**), ERK (**b**) and mTOR (**c**) and control Akt (**d**), ERK (**e**) and mTOR (**f**) molecules, with cells treated with leucine and HMB over 120 min. The data is shown as mean with SD. Significance is set at P < 0.05 and was indicted versus 0 min (*) time-point. The experiment consisted of 3 repeats all in duplicate

Having identified the impact of leucine and HMB on cell migration, the next step was to investigate their impact on relevant signaling molecules. In line with the small impact that these supplements had on aged cell migration, there was little impact on Akt, ERK or mTOR activation versus untreated aged control. Indeed, over a 2 h time course, despite small changes in signaling profiles, no significant differences were evident between untreated aged control versus leucine or versus HMB treatment (Fig. 6).

**Fig. 7:**
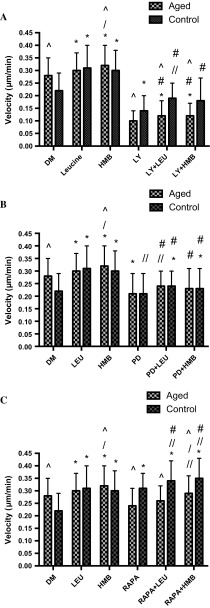
Bar charts illustrating the differences in cell velocity with the inhibition of LY294002 (**a**), PD98059 (**b**), and rapamycin (**c**) in the presence or absence of leucine and HMB over 48 h. The data is shown as mean with SD. Significance is set at P < 0.05. Significance was shown as: aged versus control (^), versus control (*), versus inhibitor (//), leucine versus HMB (/) and inhibitor with supplement versus supplement alone (#). The experiment consisted of 3 repeats all in duplicate

**Fig. 8:**
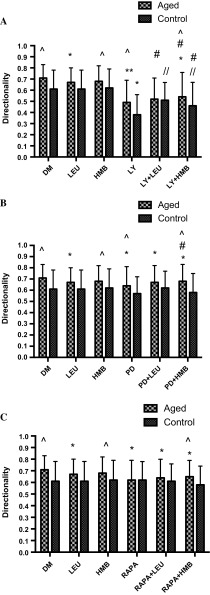
Bar charts illustrating the differences in cell directionality with the inhibition of LY294002 (**a**), PD98059 (**b**), and rapamycin (**c**) in the presence or absence of leucine and HMB over 48 h. The data is shown as mean with SD. Significance is set at P < 0.05. Significance was shown as: aged versus control (^), versus control (*), versus inhibitor (//), leucine vs. HMB (/) and inhibitor with supplement versus supplement alone (#). The experiment consisted of 3 repeats all in duplicate

**Fig. 9:**
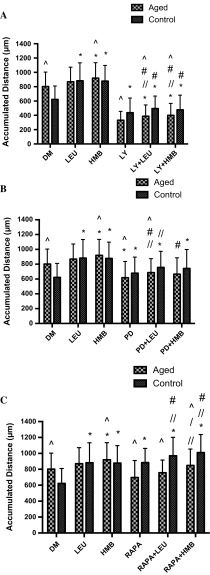
Bar charts illustrating the differences in cell accumulated distance with the inhibition of LY294002 (**a**), PD98059 (**b**), and rapamycin (**c**) in the presence or absence of leucine and HMB over 48 h. The data is shown as mean with SD. Significance is set at P < 0.05. Significance was shown as: aged versus control (^), versus control (*), versus inhibitor (//), leucine vs. HMB (/) and inhibitor with supplement versus supplement alone (#). The experiment consisted of 3 repeats all in duplicate

Given the limited impact of leucine or HMB aged cell migration or on PI3K/Akt, ERK or mTOR activation versus aged cell controls, the hypothesis to be challenged next was that these supplements would not rescue inhibited cell velocity, directionality, overall migration distance in the presence of LY294002, PD98059 or rapamycin (Fig. 7a–c), respectively. Indeed, aged cell velocity was significantly blocked by LY294002 in the absence (0.10 ± 0.04 μm/min^−1^; P < 0.001) or presence of leucine (0.12 ± 0.06 μm/min^−1^; P < 0.001) or HMB (0.12 ± 0.05 μm/min^−1^; P < 0.001) versus untreated aged control (0.30 ± 0.07 μm/min^−1^). There was no significant difference between LY294002 alone versus LY294002 with leucine or with HMB, indicating that there was no rescue of cell velocity by supplements in the presence of reduced PI3K/Akt activation (Fig. 7a). Similar to these data, when treated with LY294002 directionality was significantly reduced in the absence (0.49 ± 0.20; P < 0.001) or presence of leucine (0.52 ± 0.19; P < 0.001) or HMB (0.54 ± 0.22; P < 0.001) versus untreated aged control (0.71 ± 0.12; Fig. 8a). Finally, overall migration distance in the presence of LY294002 was significantly reduced in the absence (332 ± 123 μm; P < 0.001) or presence of leucine (388 ± 157 μm; P < 0.001) or HMB (399 ± 167 μm; P < 0.001) versus untreated aged control (802 ± 202 μm; Fig. 9a). Interestingly and in contrast to our hypothesis, there was a small but significant increase in overall migration distance, compared with LY294002, when co-incubated with either supplement (P = 0.05).

As with PI3K/Akt inhibition, when aged cells were treated with PD98059, velocity was significantly decreased in the absence (0.21 ± 0.08 μm/min^−1^; P = 0.001) or presence of leucine (0.24 ± 0.06 μm/min^−1^) or HMB (0.23 ± 0.08 μm/min^−1^), versus untreated aged control (0.28 ± 0.07 μm/min^−1^; Fig. 7b). Similarly, directionality was significantly reduced in the absence (0.64 ± 0.17; P < 0.001) or presence of either leucine (0.67 ± 0.15; P < 0.001) or HMB (0.68 ± 0.15; P < 0.001) versus untreated aged control (0.71 ± 0.12; Fig. 8b). Overall migration distance was also significantly blunted when incubated with PD98059 (618 ± 219 μm; P < 0.001) versus untreated aged control (802 ± 202 μm; Fig. 9b). Interestingly there was a small but significant increase in overall migration distance, compared with PD98059 alone, when co-incubated with either supplement (P < 0.01).

Rapamycin conditions were not significantly different (0.24 ± 0.07 μm/min^−1^) in cell velocity (Fig. 7c) versus untreated aged control (0.28 ± 0.07 μm/min^−1^) and were not altered by co-incubation with leucine (0.26 ± 0.06 μm/min^−1^) or HMB (0.29 ± 0.07 μm/min^−1^). Furthermore, rapamycin in absence (0.62 ± 0.17; P < 0.001) or presence of leucine (0.64 ± 0.16; P < 0.001) or HMB (0.65 ± 0.14; P < 0.001) significantly reduced the cell directionality versus untreated aged control (0.71 ± 0.12; Fig. 8c). The overall migrated distance in the presence of rapamycin (697 ± 213 μm; Fig. 9c) was not significantly different to untreated aged control (802 ± 202 μm), this was sustained in the presence of leucine (757 ± 159 μm) or HMB (849 ± 205 μm). Compared to rapamycin alone, the co-incubation of leucine did not significantly increase overall distance, whereas HMB was able to promote increases in overall distance (P < 0.001).

Together these data suggest that indeed the ability of leucine or HMB to rescue aged cell migration in the presence of inhibited PI3K/Akt or ERK signaling is compromised. Given the improved basal migration in control cells supplemented with leucine or HMB and the roles that PI3K/Akt and ERK appear to play in control cell migration, the next hypothesis to be tested was that these supplements may rescue inhibited cell migration in the presence of PI3K/Akt and ERK but not mTOR inhibition.

Control cell velocity was significantly reduced with LY294002 treatment, in the absence (0.14 ± 0.06 μm/min^−1^ P < 0.001) of supplements versus untreated control (0.22 ± 0.07 μm/min^−1^). In the presence of leucine, but not HMB, cell velocity was increased versus LY294002 (0.19 ± 0.06 μm/min^−1^; P < 0.001), which was not significantly different from control (Fig. 7a). The directionality of control cells was significantly blocked when inhibited with LY294002 (0.38 ± 0.18; P < 0.001) versus untreated control (0.61 ± 0.17; Fig. 8a). Co-incubation with leucine or HMB, respectively, rescued directionality versus LY294002 alone (0.51 ± 0.16; P < 0.001; 0.46 ± 0.21; P = 0.047). The co-incubation with HMB was rescued back to control, as there was no significant difference. Despite improvements in cell velocity and directionality, through co-incubation with supplements and the small increases in overall migration distance (Fig. 9a), neither leucine (495 ± 205 μm) or HMB (476 ± 206 μm) were able to significantly increase overall migration distance versus LY294002 alone (437 ± 174 μm).

Cell velocity did not change when inhibited with PD98059 (0.21 ± 0.08 μm/min^−1^) versus control (0.22 ± 0.07 μm/min^−1^); it is not therefore surprising that co-incubation in the presence of either leucine (0.24 ± 0.06 μm/min^−1^; P = 0.001) or HMB (0.23 ± 0.08 μm/min^−1^; P = 0.032) resulted in improved cell velocity versus control alone (Fig. 7b). PD98059 in the absence or presence of supplements was without impact on directionality (Fig. 8b), however when compared with control (622 ± 188 μm; Fig. 9b), migration distance was improved with PD98059 (678 ± 217 μm; P = 0.013) in the absence or presence of leucine (756 ± 217 μm; P < 0.001) or HMB (742 ± 256 μm; P < 0.001).

In control cell following rapamycin treatment, there was an increase in cell velocity (0.31 ± 0.06 μm/min^−1^; P < 0.001) versus control (0.22 ± 0.07 μm/min^−1^), which was further enhanced in the presence of leucine (0.34 ± 0.08 μm/min^−1^; P < 0.001) or HMB (0.35 ± 0.08 μm/min^−1^; P < 0.001) vs. untreated control (Fig. 7c). Indeed, when compared with rapamycin alone, leucine (P = 0.036) or HMB (P < 0.001) co-incubation both also significantly increased cell velocity versus rapamycin alone. Cell directionality was unaltered by rapamycin or by rapamycin plus either supplement (Fig. 8c), however in the presence of rapamycin, the significant increase in migration distance (884 ± 180 μm; P < 0.001) versus control (622 ± 188 μm; Fig. 9c), was further enhanced in the presence of leucine (970 ± 232 μm; P < 0.001) or HMB (1010 ± 227 μm; P < 0.001). Indeed, these increases were significantly higher than rapamycin alone for both co-incubations with leucine (P = 0.036) or HMB (P < 0.001).

**Fig. 10:**
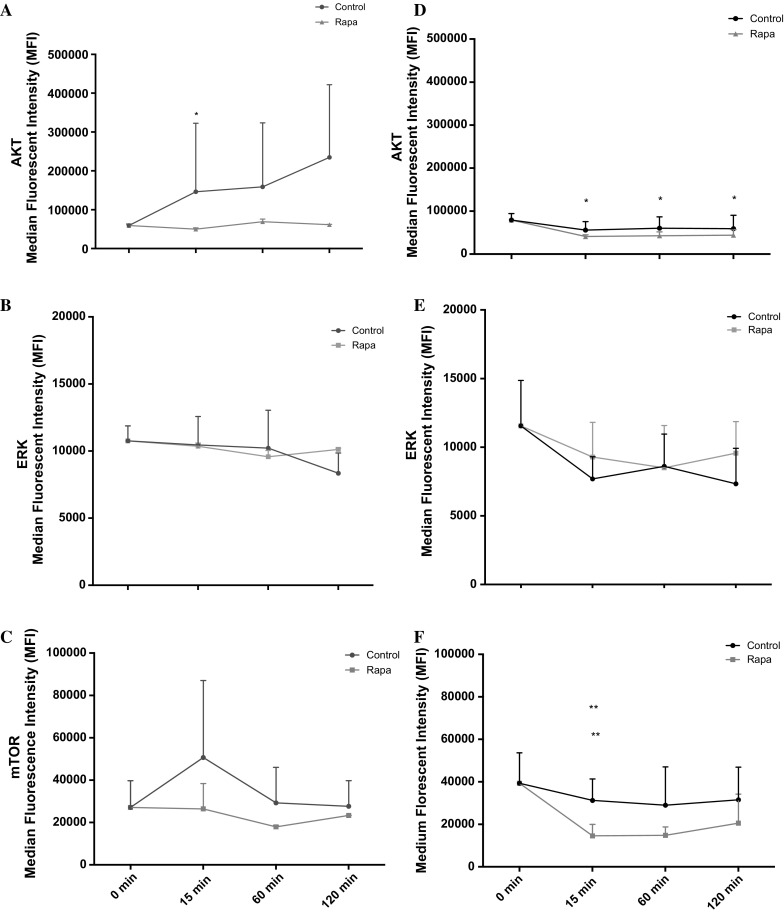
Line charts illustrating the differences in the phosphorylation of aged Akt (**a**), ERK (**b**) and mTOR (**c**) and control Akt (**d**), ERK (**e**) and mTOR (**f**) molecules, with cells treated with rapamycin over 120 min. The data is shown as mean with SD. Significance was indicted versus 0 min (*) and versus corresponding time-point (**). The experiment consisted of 3 repeats all in duplicate

Given the unexpected potential of mTOR inhibition, via rapamycin, to improve not reduce cell migration, particularly in control cells and wishing to determine possible compensatory mechanisms, which may be involved in this adaptation, the cells were incubated with rapamycin and the phosphorylation of Akt, ERK and mTOR were analysed. In both aged and control cells, rapamycin led to a significant decrease in mTOR phosphorylation. Rapamycin treated aged cells had suppressed Akt phosphorylation versus control, there was a significant decrease at 15 min (P = 0.037) versus 0 min, but not at any other time points (Fig. 10a). There was no difference between ERK (Fig. 10b) and mTOR (Fig. 10c) in aged cells until 60 min, where activity decreased in control, and increased with rapamycin. Similar to Akt, mTOR phosphorylation was greatly suppressed with rapamycin treatment versus control over 120 min. In control cells, Akt phosphorylation was also significantly reduced with rapamycin treatment at 15, 60 and 120 min versus 0 min (P = 0.005; P = 0.009; P = 0.014 respectively; Fig. 10d). The activation of ERK was reduced and suppressed until 60 min (Fig. 10e). At 120 min ERK activation spiked, which is contrary to the treatments. Rapamycin treated cells significantly reduced mTOR phosphorylation at 15 min (P = 0.026) and 60 min (P = 0.026) versus 0 min (Fig. 10f). At 120 min, the levels increase back to baseline and at 15 min, there was a significant difference between control versus rapamycin treatment (P = 0.035).

## Discussion

The aim of this study was to determine the capacity of replicatively aged versus non-replicated control cells to migrate, the signaling mechanisms underpinning this capacity and whether leucine and/or HMB could impact models of skeletal myoblast repair. We hypothesised that: (1) replicatively aged (P46–P48; “aged”) myoblasts would be less efficient at damage repair versus control (P12–P16; “control”); (2) leucine and HMB would increase the migration potential in control but not aged cells and; (3) that the PI3K and ERK but not mTOR pathways would be required for effective basal migration in both models.

The main outcome of this investigation and in contrast to hypothesis 1, was that under basal conditions, the aged passaged cells closed the wound more quickly compared to their control counterparts. This was evident in the overall velocity, directionality and accumulated distance of the cells. However, in accordance with our hypothesis 2, the supplementation of leucine or HMB enhanced the migration potential of control cells compared to the aged cells. Interestingly, the enhanced migration of control cells, in the presence of supplements, resulted in overall migration distances equivalent to those obtained in aged cells in the absence of supplements, suggesting the control cells were “catching up” with aged cells, following supplementation. Finally, in accordance with hypothesis 3, PI3K and ERK were important for basal migration in both models, but in contrast to predictions, mTOR inhibition, not activation, enabled facilitated wound repair in the absence or presence of supplements and most markedly in control versus aged cell models.

To date, when studying the impact of age and nutrition on muscle adaptation, the majority of published research has focused on sarcopenia (Churchward-Venne et al. 2014), the progressive loss of muscle mass and strength with age and on anabolic resistance in aged individuals (Burd et al. 2013). However, it is also reported that the capability of the muscle to regenerate in the aged individuals is impaired, compared with control counterparts (Jang et al. 2011). In vitro, mechanistic studies to underpin this latter observation are, however, currently sparse. Owens et al. (2015) investigated the impact of vitamin D on muscle regeneration in primary human muscle cells, and reported average control migration rates equivalent to those in this model (666 ± 288 vs. 622 ± 188 μm). Our data challenge our hypothesis of impaired migration with age, with aged cells migrating more efficiently than control ones, perhaps as a result of improved PI3K/Akt and ERK signaling. The improved wound closure is not a result of cellular replication, with previous studies using mitomycin-C to block proliferation in unaged and aged myoblasts reporting migration capabilities which remain intact (Falcone et al. 1984; Dimchev et al. 2013). Replicatively aged C_2_C_12_ myoblasts also retain telomeres (Yaffe and Saxel 1977; Holt et al. 1996; O’connor et al. 2009) and express decreased levels of IGF-1 and Akt phosphorylation (Benbassat et al. 1997; Léger et al. 2008), similar to aged primary muscle stem cells. The absence of a response to leucine and HMB supplementation in aged cells may arise as a result of optimised migration under basal conditions—this is supported via the observation that when control cells are supplemented with either leucine or HMB, migration is significantly improved, but only to levels seen in untreated aged cells.

To substantiate this theory, in both cell models, when the PI3K/Akt pathway was inhibited, the cell velocity, directionality and accumulated distance all decreased, suggesting that this pathway is integral to myoblast migration. The basal enhancement in the aged versus control cells may account for improved migration under control conditions. Neither leucine nor HMB were able to rescue reduced migration in the presence of LY294002, suggesting the fundamental role of this pathway in effective migration of myoblasts. Interestingly, while PD98059 reduced basal velocity and distance migrated in aged cells, it was without impact in control cells extending our observations to suggest that PI3K and ERK function together to facilitate increased migration in aged versus control cells. Under inhibitor conditions and unlike PI3K inhibition, co-incubation with leucine or HMB incurred partial rescue to the aged cell velocity and migration distance. By contrast, while basal velocity and overall migration distance were not negatively impacted by PD98059 in control cells, the facilitated migration velocity and distance in the presence of leucine or HMB was significantly reduced, compared with either supplement alone. Therefore, while partial rescue can be incurred via supplementation in aged cells when ERK is inhibited, the capacity of control cells to respond to supplements is reduced in the absence of ERK activation, despite no impact on basal cell migration potential. These data suggest both shared and divergent pathways underpinning aged versus control myoblast migration.

Dai et al. (2015) recently researched the effects of leucine on rat satellite cell proliferation and differentiation. The authors reported that leucine promoted proliferation and differentiation through the mTOR-MyoD signalling pathway. Research reinforcing these results demonstrated that leucine starvation led to inhibition of myoblast differentiation (Averous et al. 2012). Areta et al. (2014) also demonstrated the benefits on supplementing leucine on C_2_C_12_ muscle cell growth. Research by Kornasio et al. (2009) is the only study, to the author’s knowledge, to investigate the effects of HMB on aged myoblast activity. This study suggested that HMB stimulated myoblast proliferation, differentiation and survival compared to control, with both the PI3K/Akt and ERK/MAPK signalling pathways involved in these processes. Cell migration was not assessed. Further, studies by Kornasio et al. support the concept that HMB has beneficial effects on the proliferation and differentiation of myoblasts, through the MAPK/ERK and PI3K/Akt pathways (Kornasio et al. 2009; Vallejo et al. 2016). Although migration in the presence of supplements was not investigated in these reports, data from Dimchev et al. suggest that the ability of myoblasts to migrate depends on the PI3K/Akt and ERK/MAPK pathways (Dimchev et al. 2013).

The data from our study support the hypothesis that when inhibiting the PI3K/Akt pathway with LY294002, the cell velocity, directionality and accumulated distance is reduced. Previous studies have supported this finding (Suzuki et al. 2000; Kawamura et al. 2004; Al-Shanti et al. 2011; Dimchev et al. 2013). Raftopoulou and Hall (2004) suggested that the PI3K/Akt pathway is integral in the development of cellular protrusions. Moreover, Kawamura et al. (2004) demonstrated that the PI3K/Akt pathway is essential for cell migration through lamellipodial formation, which is important in cell directionality and velocity (Raftopoulou and Hall 2004). It is apparent therefore, that the PI3K/Akt pathway is integral for myoblast migration, irrespective of the replicative status of the myoblasts. This may not be the case for ERK activation, where ERK activity appears important for improved migration of the aged cells, but not control, in this study. However, the data underpinning a role for ERK in myoblast migration are equivocal. For instance, Leloup et al. (2007) suggested that the ERK/MAPK pathway was responsible for stimulating growth factor mediated stimulation of myoblast migration. Conversely, Ranzato et al. (2009) suggested that it was primarily Akt and p38 MAPK signaling proteins that stimulated myoblast migration not the ERK pathway. Whereas, other research has also supported the notion that the ERK/MAPK pathway was involved in cell migration (Al-Shanti et al. 2011; Dimchev et al. 2013). The differences in studies could be attributed to the dose of inhibitor used as well as the myoblast models, alternatively, this apparent controversy warrants further investigation, as it may have important implications for cellular need, for example, fusion in the control versus migration in the aged models.

Intriguingly, and not reported in the Kornasio study (Kornasio et al. 2009), when rapamycin was added to the wound model detailed in this manuscript, it was with no impact on basal migration of aged cells, but significantly increased, not decreased, cell velocity and basal migration of control cells, which were both further increased with co-incubations of leucine or HMB. The mTOR pathway is largely regarded to be integral for muscle protein synthesis (MPS) and is stimulated by both exercise and protein ingestion, and is further increased when both are combined (Hawley et al. 2011). Further research proposes that leucine is the primary initiator of MPS (Churchward-Venne et al. 2012). The mTOR target is downstream of the PI3K and Akt signaling cascades which then stimulate S6K which leads to MPS (reviewed in: (Sharples et al. 2015). The impact of this pathway on muscular hypertrophy and muscle wasting is well known (reviewed in: Sharples et al. 2015), however, the impact on cell migration has not been studied. These observations would suggest that mTOR activation is detrimental to control cell migration, but integral to myoblast hypertrophy/fusion (Averous et al. 2012; Areta et al. 2014; Dai et al. 2015), again raising the concept of differential drivers of cellular behavior with age. Indeed, the data derived in this study suggest that the PI3K/Akt pathway is an integral pathway, shared in control and aged cell migration, that ERK further enhances aged cell migration, but that mTOR reduces control cell migration. These changing signaling pathways may underpin the adaptation that appears to have occurred as a result of replicative ageing.

## Conclusion

Taken together, basally, the aged cells migrate more quickly than control myoblasts, potentially at the expense of efficient fusion (Sharples et al. 2011). However, with the supplementation of leucine and HMB, the capacity of the control cells to migrate is increased to that of the aged cells. This implies that 1, the control cells are more responsive to the supplements or 2, that maximal migration capacity has been attained in aged cells under basal conditions and therefore supplements cannot improve their migration capability. In control myoblasts, it is the PI3K/Akt pathway that appears central for migration, which is in line with previous research. However, our data show that the ERK/MAPK pathway is less important for control myoblast migration, where the results in the literature are still equivocal. Activation of both pathways are required for replicatively aged myoblast migration. To the author’s knowledge, this is the first study to investigate the potential role of the mTOR pathway in myoblast migration. The results are interesting, showing that this pathway, when activated, could reduce control myoblast migration, perhaps to ensure successful protein synthesis and hypertrophy. Differential activation of the PI3K/Akt, ERK/MAPK and mTOR pathways appear to underpin the differences observed in control or supplemented control and aged myoblast migration. Future research is required to establish further the mechanisms underpinning altered migration, including the roles of lamellipod/filapod formation and actin polarisation; cell/matrix interactions should also be determined.
